# Effects of season long participation on ACL volume in female intercollegiate soccer athletes

**DOI:** 10.1186/s40634-019-0182-8

**Published:** 2019-03-28

**Authors:** Karen M. Myrick, Andreas Voss, Richard S. Feinn, Thomas Martin, Bernadette M. Mele, Juan C. Garbalosa

**Affiliations:** 1 Quinnipiac University, School of Nursing, Hamden, CT USA; 2 Department of Trauma Surgery, University Hospital Regensburg, Franz-Josef-Strauß-Allee, 11 93053 Regensburg, Germany; 3 Department of Orthopaedic Sports Medicine, Technical University of Munich, Munich, Germany; 4 Quinnipiac University, School of Medicine, Hamden, CT USA; 5 Quinnipiac University, School of Health Sciences, Hamden, CT USA

## Abstract

**Background:**

The aim of this study was to characterize the volumetric changes of the anterior cruciate ligament over the course of a competitive soccer season in female athletes.

**Methods:**

Seventeen Division-I collegiate soccer players were recruited. Two data collection sessions were conducted. The first data collection occurred prior to the start of the soccer season. Each subject completed a brief questionnaire, had height and weight measured, underwent a clinical assessment of their anterior cruciate ligaments and an eight sequence magnetic resonance imagery of their knees. Contours of the anterior cruciate ligaments were outlined in sagittal T-2 weighted MR images and volumes were calculated using Medical Image Processing, Analysis, and Visualization software. Presence or absence of edema within the ligament was determined in pre and post season scans. All subjects were followed during the season to determine if a lower extremity injury had been sustained.

**Results:**

Mean ligament volume significantly increased from preseason to postseason (p=.006). There was a 10% increase in the percentage of knees with edema pre to post season.

**Conclusions:**

The physical demand of a competitive soccer season in female collegiate athletes appears to cause an increase in volume of the anterior cruciate ligament. The increase in volume may be related to the accumulation of microscopic tears over the course of the season which induce inflammation and edema. The volumetric changes in the ligament may have significant clinical implications, however further studies must be done to determine the relationship between anterior cruciate ligament volume and risk of injury.

## Background

Anterior cruciate ligament (ACL) injuries are one of the most common injuries that occur in competitive sports (Alentorn-Geli et al. 2009; Griffin et al. 2000). Seventy percent of ACL injuries are sports related, affecting female athletes two to ten times more than males (Arendt and Dick 1995; Ireland 2002). Injury rates as high as 2.8 and 3.2 injuries per 10,000 athlete exposures have been reported in women’s collegiate sports (Mihata et al. 2006).

These devastating injuries are associated with a lengthy and often painful rehabilitation, psychological effects, and severe financial burdens with the average cost associated with each injury totaling $17,000 (Hewett et al. 2009; Hewett et al. 2005). Besides the short-term and financial burdens, long-term health complications such as an increased risk of meniscal tears and the development of early onset post-traumatic osteoarthritis are a major concern for individuals with ACL injuries regardless of whether or not ACL reconstruction was performed (Lohmander et al. 2004). In women 12 years post ACL tear, roughly 50% of individuals had radiographic evidence of osteoarthritis with approximately 75% of those women claiming that they had knee issues that affected their quality of life. Both long-term physical consequences and high economic costs of ACL injuries stress the importance of the need to better understand the mechanism of injury and the development of effective preventive programs.

There are many factors associated with the increased risk of females sustaining an ACL injury including, but not limited to, improper lower limb joint biomechanics, type of athletic maneuver such as cutting/landing, hormone levels and anatomical and structural factors. While previous studies have correlated ACL volume with injury retrospectively, little information is available on how the ACL volume changes during periods of intense physical activity. The aim of this study was to characterize the volumetric changes of the ACL over the course of a competitive soccer season in female athletes.

## Methods

A sample of 17 Division-I female collegiate soccer players between the ages of 18 and 23 were recruited for participation in this study. To be included in the study, subjects must have been members of the Quinnipiac University women’s soccer team without previous history of ACL injury, and without any musculoskeletal, cardiovascular, or neuromuscular condition that would be contraindicated for participation in this study. Subjects were excluded if they had any type of metal present in their body or were claustrophobic. The study was approved by the University’s Internal Review Board, and all participating subjects gave consent to this study.

The study consisted of two data collection sessions lasting approximately 75 min each. The first data collection session occurred prior to the start of the 2013–2014 soccer season, at which time each subject completed a brief questionnaire, had height and weight measured, and underwent a bilateral clinical assessment of the integrity of their ACLs.

Prior to undergoing the imaging procedure, all subjects completed an MRI Procedure Screening Form. Subjects then underwent an MRI examination of their right and left knees. Each subject was thoroughly screened for safety by a qualified radiologic technologist trained in the use of the MRI unit (Toshiba Vantage 1.5 Tesla Magnetic Resonance Imaging, Toshiba America Medical Systems, Inc., Irvine, CA). The subject was positioned supine, feet directed first into the magnet; the knee to be imaged was supported to ensure no movement, and then placed in a fully extended position with the lower extremity in 15 to 20 degrees of external rotation. A quadrature imaging coil specific for knee imaging was used. The knee joint being imaged was placed at the isocenter (1/2 in. inferior to the patella) of the knee. Table 1 lists the sequences, imaging techniques, and timing parameters used to obtain the images.

**Table 1:** MRI sequences

Protocol	Time (min:sec)	Pulse Sequence	Time of Repetition (ms)	Time of Echo (ms)
Coronal Locator	0:21	Fast Spin Echo (8) 2D	1112	15
3 Plane Locator	0:30	Field Echo 2D	72	9
Shimming	0:13	Field Echo 2D	200	4.8/9
Sagittal Proton Density	4:30	Fast Spin Echo (4) 2D	2750	18
Sagittal T2 Weighted Fat Sat	3:46	Fast Spin Echo (7) 2D	4092	60
Coronal T2 Weighted	3:46	Fast Spin Echo (7) 2D	4092	60
Coronal Proton Density	4:23	Fast Spin Echo (4) 2D	2300	18
Coronal STIR (Short Tau Inversion Recovery)	3:27	Fast Spin Echo (7) 2D	3750	48
Coronal T2 Weighted 2 .5mm	3:18	Fast Spin Echo (7) 2D	3596	60
Sagittal T2 Weighted 2 .5mm	3:18	Fast Spin Echo (7) 2D	3596	60

In addition, the field of view (FOV) that was maintained for each knee was 16 × 16 mm within a coarse matrix (192 × 256). A slice thickness of 3 mm with a 30% slice gap (0 .9mm) was used for the first 5 scans of each knee. For the remaining 2 scans, a slice thickness of 2.5 mm with a 30% slice gap (0 .7mm) was used for each knee. The total approximate time for the subject in the scanner was 60 min for imaging of both knees.

Within 2 weeks of the completion of the season, each subject returned for a second data collection session. During the post-test, weight was re-measured and the subjects underwent a follow-up MRI examination of their knees, bilaterally, using the previously stated procedures.

**Fig. 1:**
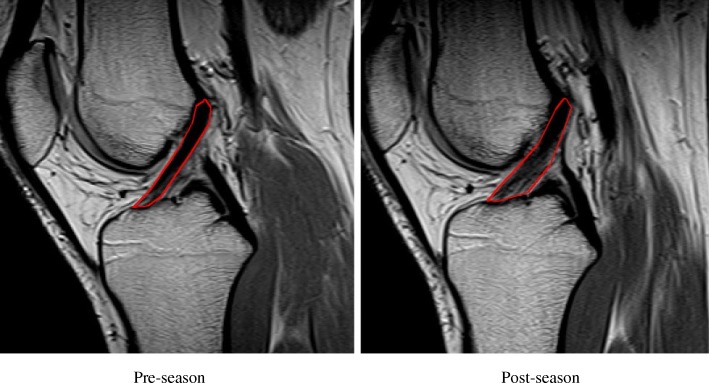
MRI Image of Same Knee at Pre and Postseason

Contours of the ACL were manually outlined in sagittal T-2 weighted MR images (see Fig. 1) and volumes were calculated using the Medical Image Processing, Analysis, and Visualization (MIPAV, National Institutes of Health, Bethesda, USA), which has been shown to be a valid method of determining structural properties of the ACL in vivo *(*Chaudhari et al. 2009*)*. All subjects were followed during the season to determine if a lower extremity injury had been sustained.

At the completion of the competitive season, the subjects were asked to return for a second data collection session. Sixteen of 17 athletes were available for follow-up. At this time, the subjects’ weight was re-measured after which they underwent a follow-up MRI examination of their right and left knees using the previously stated procedures.

Presence or absence of edema within the ACL was determined in pre and post season scans by an orthopedic surgeon, which were randomly allocated during the measurement.

Descriptive statistics to characterize the study group were calculated using means and standard deviation. To test for change in ACL volume from pre-season to post-season a multilevel linear mixed model with time point (pre and post) nested within knee (right and left) further nested within subject was run. The model included random effects for knee within subject and subject, and a fixed effect for time point. A general estimating equation using a binomial distribution with logit link was used to test for change in occurrence of edema from pre- to post-season. Follow-up analyses had training load entered as a covariate and the interaction between training load and time point tested if training load during the season was predictive of change in volume or change in edema. The alpha level for all comparisons was set at 0.05 and analyses were performed using SPSS v24.

## Results

**Fig. 2:**
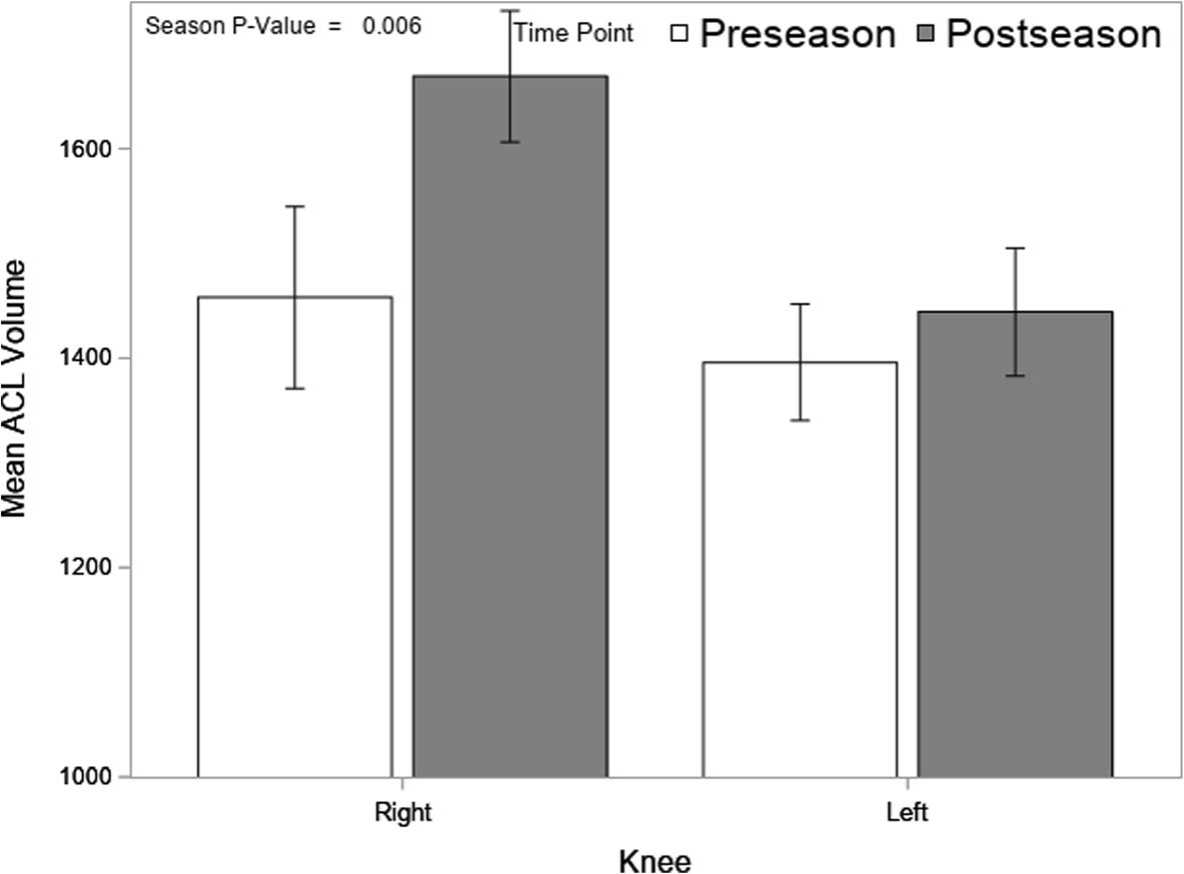
Mean (± SE) ACL Volume by Knee and Season

The sample was made up of 17 subjects who were, on average, 19.7 years of age, 167 cm tall, 63.9 kg and had a BMI of 22.8. Table 2 shows demographic characteristics of the sample. Mean ACL volume significantly increased from preseason to postseason (*p* = .006). Averaged across both knees mean volume was 1426 cc (SD = 288) at preseason and 1556 cc (SD = 269) at postseason. Figure 2 shows mean volume by knee and time point. There was greater volume increase in the right knee than the left and the difference between knees was significant (*p* = .047), where the right knee had an average increase of 211 cc versus an increase of 48 cc for the left.

**Table 2:** Subject Demographics

Characteristics	Mean	Std Deviation
Age	19.7	1.0
Height (cm)	167	3.5
Weight (kg)	63.9	6.8
BMI	22.8	2.3

**Fig. 3:**
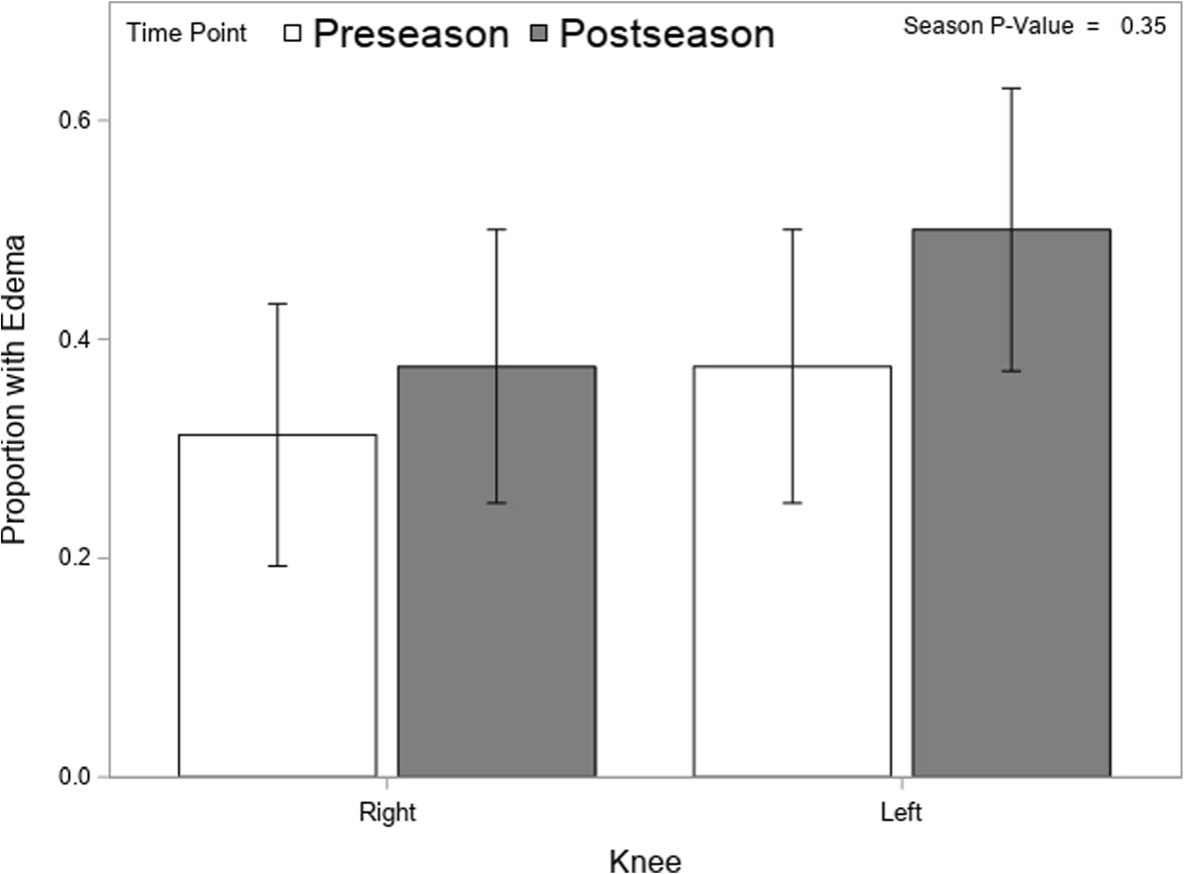
Proportion (± SE) of Athletes Displaying Edema by Knee and Season

Figure 3 shows the proportion of MR images showing the presence of edema. There was an increase in the percentage of knees with edema from 34% at preseason to 44% at postseason, but the difference was not statistically significant (*p* = 0.35). It should be noted the change in edema was not consistent; 13 of the 32 knees changed status, of which 8 went from no edema at preseason to edema at postseason and the other 5 went in the opposite direction. To assess the relationship between change in ACL volume and change in edema a multivariate mixed model with volume and edema as outcomes resulted in a nonsignificant small positive correlation (r = 0.19) between the changes. Interestingly, the right knee showed greater ACL volume while the left knee had a higher incidence of edema.

## Discussion

To the authors knowledge this is the first study to examine the effects of a pre-to-post season comparison of ACL volume in competitive athletes. Our most important finding was that in the cohort of Division-I female soccer athletes, we observed a significant increase in the mean ACL volume after the competitive season compared to pre-season. In addition, an increase in the occurrence of edema of the ACL in post-season scans was noted in this study (Fig. 2), (*p* = 0.35). Increase in volume was weakly associated with occurrence of edema.

This is a unique study that demonstrates the volumetric changes that occur in the ACL in response to training. We hypothesize that repetitive subacute trauma occurring over the course of the competitive soccer season leads to microscopic tears in the ligament inducing an inflammatory response and subsequent remodeling of the ACL which results in increased volume.

It is known that injured ligaments heal in 3 consecutive stages: (1) acute inflammatory stage, (2) proliferative regenerative/repair stage, and (3) remodeling phase (Hauser et al. 2013). It’s likely that the post season scans in our study represent ACLs that have been damaged over the course of the season and are in one of these stages of healing, namely, the proliferative regenerative/repair stage, which represents fibroblast proliferation and rebuilding of the ligament tissue matrix (Frank 2004). While it is known that physiologic remodeling of an injured ligament causes functional changes (Frank et al. 1995; Frank et al. 1999; Niyibizi et al. 2000; Shrive et al. 1995; Woo et al. 2006), it is unclear if the volumetric changes seen in the ACLs in our study correspond to increased risk for injury since none of the subjects in our study incurred an ACL injury during the soccer season, and laxity was not measured in an objective manner.

The structural integrity of biomaterials depends on complex interactions between the individual material and their surroundings. At the extreme of the loss of such integrity, the tissue can fail, but the subtler tissue responses that occur in the macroscopic sub failure regime are also critical in the path mechanisms and physiological dysfunction.

(Zhang et al. 2016). In ligaments and tendons, type I collagen is the principal structural element of the extracellular matrix, which acts to transmit forces between bones or bone and muscle. The characterization of collagen fibril morphology and reorganization is essential to understanding how tissues develop and obtain mechanical attributes (Provenzano and Vanderby 2006). Understanding changes in mesoscale collagen fiber networks can improve the detection and prediction of local tissue failure, because the eventual rupture of collagenous tissue involves damage to collagen fibre bundles and/or a large number of distributed collagen fibers (Beveridge et al. 2017). Collagen re-organization is of interest, and the T2-weighted MRIis a measure of magnetic resonance signal relaxation that is related to the degrees of free water bound by collagen (Woo et al. 2006).

A secondary finding of our study revealed that across the season, athletes experienced greater ACL volume changes in their right knee, while also demonstrating an increase incidence of edema in their left knee. This finding could be potentially explained by leg dominance. All 17 subjects in the current study identified as being right leg dominant (i.e kicking the ball with their right foot). Previous studies have demonstrated that the plant leg during a kicking maneuver receives considerable stress at the knee joint and could be a possible explanation for the increased edema seen in this extremity across the season. It is also possible, that the external forces that are placed on the kicking limb during contact with the ball could cause additional repetitive subacute trauma on the ACL contributing to the edema. (Barfield 1998; Nunome et al. 2002).

In future studies we would like to correlate volumetric changes of the ACL with structural properties by objectively measuring ACL laxity pre-season and post-season using a KT-1000 arthrometer. We also hope to follow up on our subjects for a longer period of time to determine if the ACL volumes return to baseline after a given rest period. We would also like to see if the same changes occur in different demographic groups including males and adolescents.

Limitations include low statistical power. With an N of 17, the power of the study may not have been high enough to reach statistical significance, and to demonstrate a change in edema at pre and post season. The study was not blinded, and therefore there may have been a biased response calculated into the results. There was no objective clinical data to measure edema.

## Conclusion

The intense physical demand of a competitive soccer season in female collegiate athletes appears to cause an increase in volume of the ACL. The increase in volume of the ACL in this study may be related to the accumulation of microscopic tears over the course of the season which induce inflammation and edema of the ligament resulting in an increase in ligament volume. The volumetric changes seen in the ACL may have significant clinical implications, however further studies must be done to determine the relationship between ACL volume and injuries.

## Abbreviations

ACL: Anterior cruciate ligament
FOV: Field of view
MR: Magnetic resonance
